# Understanding changes and stability in the long-term use of technologies by seniors who are aging in place: a dynamical framework

**DOI:** 10.1186/s12877-019-1241-9

**Published:** 2019-08-28

**Authors:** S. T. M. Peek, K. G. Luijkx, H. J. M. Vrijhoef, M. E. Nieboer, S. Aarts, C. S. van der Voort, M. D. Rijnaard, E. J. M. Wouters

**Affiliations:** 1 School of Social and Behavioral Sciences, Department of Tranzo, Tilburg University, Tilburg, The Netherlands; 2 Institute of Allied Health Professions, Chair of Health Innovations and Technology, Fontys University of Applied Sciences, Eindhoven, The Netherlands; 3 Panaxea b.v, Amsterdam, the Netherlands; 4 Department of Patient & Care, Maastricht University Medical Center, Maastricht, The Netherlands; 5 Department of Family Medicine and Chronic Care, Vrije Universiteit Brussel, Brussels, Belgium

**Keywords:** Dynamical systems theory, Longitudinal qualitative research, Aging in place, Technology acceptance, Technology adoption, Smart home, E-health, ICT, Gerontechnology, Consumer appliances

## Abstract

**Background:**

If technologies are to support aging in place, then it is important to develop fundamental knowledge on what causes stability and changes in the use of technologies by seniors. However, longitudinal studies on the long-term use of technologies that have been accepted into the home (i.e., post-implementation use) are very scarce. Many factors potentially could influence post-implementation use, including life events, age-related decline, changes in personal goal orientation, and various types of social influences. The aforementioned factors are likely to be interrelated, adding to the complexity. The goal of this study is to better understand changes and stability in the use of technologies by independent-living seniors, by using a dynamical systems theory approach.

**Methods:**

A longitudinal qualitative field study was conducted involving home visits to 33 community-dwelling seniors in the Netherlands, on three occasions (2012–2014). Interviews were held on technology usage patterns, including reasons for stable, increased, declined and stopped use. Technologies were included if they required electric power in order to function, were intended to be used in or around the home, and could support activities of daily living, personal health or safety, mobility, communication, and physical activity. Thematic analysis was employed, using constant case comparison to better understand dynamics and interplay between factors. In total, 148 technology use patterns by 33 participants were analyzed.

**Results:**

A core of six interrelated factors was closely linked to the frequency of technology use: emotional attachment, need compatibility, cues to use, proficiency to use, input of resources, and support. Additionally, disruptive forces (e.g., social influences, competition with alternative means, changes of personal needs) could induce change by affecting these six factors. Furthermore, long-term technology use was in some cases more resilient to disruption than in other cases. Findings were accumulated in a new framework: Dynamics In Technology Use by Seniors (DITUS).

**Conclusions:**

Similar to aging, the use of technologies by older people is complex, dynamic and personal. Periods of stability and change both occur naturally. The DITUS framework can aid in understanding stability and instability of technology use, and in developing and implementing sustainable technological solutions for aging in place.

**Electronic supplementary material:**

The online version of this article (10.1186/s12877-019-1241-9) contains supplementary material, which is available to authorized users.

## Background

People are living longer and, in many cases, healthier lives than ever before. Simultaneously, the number of older persons is growing faster than the number of people in the traditional working ages in many countries, leading to pressure on health systems, and an increasing demand for care, services and technologies to prevent and treat chronic diseases and conditions [1]. A common policy response is to encourage ‘aging in place’, which can be defined as *“remaining living in the community, with some level of independence, rather than in residential care*” [2]. As a consequence of these developments, an increasing number of adults are destined to live independently for a longer period of time. For example, in the Netherlands already almost 95% of all senior citizens live independently [3]. Still, aging in place can be challenging for older adults, and views on the ideal way to age in place may differ between older adults [4].

More and more, technology is viewed as a potential resource for facilitating or improving aging in place [5, 6]. Technologies for aging in place are typically designed to support or enhance activities of daily living, personal health or safety, mobility, communication, and physical activity [7]. They are also referred to as gerontechnology, ambient assisted living technology, smart home technology, or eHealth. Specific examples include vital signs monitoring and fall detection devices, mobile phones specifically designed for seniors, and medication reminders [7, 8]. Additionally, older adults can take benefit of generally available consumer appliances and devices that play a role in staying independent, active and healthy (e.g., fitness equipment to stay physically active, home appliances for activities of daily living, and information and communication technologies to support social contact). It could be argued that an older adult’s daily life and participation in society is, to a large extent, influenced by the use of these widely available types of technology [9, 10]. Yet, all aforementioned technologies can only provide benefits if they are used by older adults. In this respect, it is important to acknowledge that successful aging in place is essentially a matter of adapting to aging and environmental changes [11, 12]. For technology to play a role in independent living, it is important to develop fundamental knowledge on what causes stability and changes in the use of technologies by seniors over time. Preferably, the use of supporting technology is sustainable. At the same time, surprisingly little research has been conducted on what inhibits and promotes the sustained use of technologies by seniors.

Within the scientific literature, the emphasis very much lies on why independent-living older adults would start to use technology in the first place (i.e., pre-implementation acceptance) [7]. This also applies to existing technology acceptance models [13]. Studies on (fluctuations of) the use of technologies that have been accepted into the home (i.e., post-implementation acceptance) are very scarce. In particular, longitudinal studies are lacking [7, 14]. Additionally, the majority of studies only focus on the acceptance of one (type of) technology, thereby neglecting the fact that the use of a particular technology may very well be dependent on the availability and use of technological and non-technological alternatives [9, 15]. Furthermore, many more factors potentially could influence why older adults would continue or change the use of technologies in the home. These include the occurrence of life events, age-related decline, changes in personal goal orientation, technological changes and various types of social influences [16–19]. The aforementioned factors are likely to be interrelated, adding to the complexity.

In aiming to understand changes and stability in frequency of use of technologies over time, Dynamical Systems Theory (DST) can be of use [20]. DST stems from the fields of mathematics and physical sciences and is increasingly applied in other fields including biology and psychology [20, 21]. It has generated interest and excitement as a series of principles and tools for studying change and equilibria (i.e., states of stability) [22, 23]. In DST, values of variables at one time are modeled as functions of those same variables at earlier times. In contrast to linear (non-dynamical) models, variables can serve as both dependent and independent variables at the same time. This is why feedback loops play an important role in dynamical system models [22]. Together, one or more feedback loops of variables form a ‘system‘of interacting components. The current state of a system can be challenged by external disturbances and internal fluctuations. Ultimately, these disturbances and fluctuations can lead to a breakpoint (i.e., the point at which a system shifts to an alternative state) [24]. DST can be used to simulate or test mathematical equations of change, or it can be used as a metaphor, whereby concepts are applied qualitatively without the use of mathematical relationships [20, 25]. In the current qualitative study, DST is used as a theoretical lens while addressing the following research questions: (1) When and why does the frequency of use of technology by independent-living older adults remain stable over time; and (2) What drives changes in the frequency of use of technology by independent-living older adults. In this pursuit, the current study was designed to include technologies that are used in or around the home, require electric power, and can support activities of daily living, personal health or safety, mobility, communication, and physical activity. Furthermore, we aimed to include technologies that independent-living older adults have much experience with (e.g., home appliances), as well as technologies that may be more novel to them (e.g., ICT devices, assistive technology) [26]. As suggested by others, we will illustrate our findings by using graphical representations of DST concepts [21, 22].

## Methods

### Design

The current study was set up as a prospective longitudinal qualitative field study [27], involving home visits to independent-living older adults on three occasions (*t*_1_, *t*_2,_ and *t*_3_; 2012–2014). The longitudinal field study led to the publication of two papers: a paper solely focused on how and why technologies are acquired by independent-living older adults [26], and the current paper that focuses on the long-term use of technologies after acquirement. The methodology of both papers partly overlaps.

### Sampling

After receiving approval for the study from the Ethics Review Board of the Tilburg School of Social and Behavioral Sciences, a purposive sample of independent-living older adults with different health statuses, living arrangements, and levels of technology experience was recruited. Participants were recruited in a medium-sized city in the Netherlands via two home care providers, a senior volunteer organization, a local tablet computer pilot project, a local shopping center, and word of mouth contacts. Criteria for the inclusion of participants were: (1) independently living at home, (2) aged 70 years or older, (3) Dutch nationality, and (4) no cognitive impairment as measured by the Mini-Mental State Examination (MMSE) [28] using a score of 24 as cutoff [29]. It was decided to include individuals aged 70 or older, because older age is related to an increased difficulty to continue to age in place [30, 31]. Potential participants were given an information letter and were telephoned to schedule the first home visits if they were interested in participating. Of the 72 individuals approached, 53 agreed to participate (*N* = 53, *t*_1_). One participant was included per household. Subsequently 18 and 2 participants dropped out (*N* = 35, *t*_2_; *N* = 33, *t*_3_). Reasons for drop out were: not interested in continuing (*n* = 5), deceased (*n* = 4), somatic health problems (*n* = 4), cognitive impairment (*n* = 2), too busy providing informal care for their partner (*n* = 2), no longer living independently (*n* = 2), and lost contact (*n* = 1). For the study reported here, only individuals who participated in *t*_1_, *t*_2_ and *t*_3_ were included (*N* = 33).

### Data collection

Pairs of researchers (SP, MN, SA, CvdV, and MR) performed home visits. SP, SA and MR all have a background in psychology, while MN and CvdV both have a background in healthcare. Informed consent was acquired at the start of each first visit. At the end of the first visit, participants were offered a magazine subscription of their choice. Prior to subsequent visits, participants were sent a letter containing information on the research project’s progress and called to schedule a visit at their convenience.

At *t*_1_ (September – December 2012) the aim was to gain an initial understanding of participants’ lives, their perceptions and attitudes towards technologies, and their use of technologies. Three types of data collection were performed: (1) background information on educational level, civil status, living arrangement, level of formal and informal care, chronic conditions, subjective health status, occurrence of life events in the last 12 months, frailty as measured by the Tilburg Frailty Indicator (TFI) [32], and cognitive functioning as measured by the MMSE [28]. TFI scores could range between 1 and 15, MMSE scores could range between 0 and 30; (2) an inventory of technologies in the home. For this purpose, participants and researchers jointly made a tour through the home. Frequencies of use of these technologies were recorded using the categories: (nearly) daily; at least once a week; at least once a month; less than once a month, and stopped using, or never used; (3) semi-structured interviews in which participants were interviewed on reasons for the frequency of use of technologies. A topic list was adjusted as data collection progressed (see Additional file 1 for the interview guide that was used).

At *t*_2_ (May – July 2013) and *t*_3_ (March – June 2014) data collection was aimed at understanding why participants’ use of technologies remained stable or changed since *t*_1._ First, the same type of background information on participants as in *t*_1_ was gathered, and the inventory of technologies in the home was updated. Second, semi-structured interviews were conducted on at least one technology of which the frequency of use was identical to the previous visit, at least one technology of which use had increased, and at least one technology of which use had decreased or stopped entirely. Which technologies were discussed depended on preferences of the participants (who displayed strong feelings towards certain technologies) and on suggestions by the researchers (who aimed to understand the usage of multiple types of technology). During the interviews, we took into account background information that was gathered on each participant and relevant themes which had emerged in previous interviews. We made sure that at least one of the two visiting researchers had visited the participant before. The topic list used was further evolved as data collection progressed. All of the interviews were audiotaped and transcribed verbatim.

### Analysis

In total, 148 technology use patterns by 33 participants were analyzed. Analysis took place during and between all three waves of the data collection and was supported by the use of qualitative data analysis software (Atlas.ti). SP, KL, MN, SA, CvdV, and MR employed realist thematic analysis, which focuses on reporting experiences, meanings and the reality of participants [33, 34].

The thematic analysis entailed three stages: open coding, axial coding, and selective coding. For open coding, we studied transcripts and attached inductive codes to quotations relevant to the research questions. All *t*_1_ transcripts, two-thirds of the *t*_2_ transcripts, and one-third of the *t*_3_ transcripts were first coded independently by two different researchers. The two researchers then discussed their analyses and produced a single coded version of each transcript. Coding was detailed; often multiple codes representing different factors influencing technology use were attached to quotations. During and after each wave, axial coding was conducted. In this pursuit, the open coded transcripts were combined into one Atlas.ti file by SP. This file was used in group sessions in which similar codes were merged, and overarching themes were formed.

After the open and axial coding of the *t*_1_ transcripts, few new codes were added during the analysis of the *t*_2_ and the *t*_3_ transcripts, which indicated that data saturation was reached with regards to which factors and themes played a role in explaining the frequency of use of technologies. Subsequently, selective coding was used in order to better understand the dynamics and interplay between these factors and themes over time.

During selective coding, SP applied constant case comparison [35], systematically comparing the use of various types of technology by each participant, and between participants. To facilitate this process, SP created a matrix of each of the 33 participants’ longitudinal qualitative data [36]. As such, each matrix included the factors and themes that had played a role in the participant’s use of technologies at *t*_1,_ *t*_2_ and *t*_3._ Analysis of the matrixes informed the iterative shaping of a comprehensive framework of our findings. DST was used as a theoretical lens during this process, specifically to further refine our understanding of periods of stable and changing use. In the abovementioned analytical process, insights and findings were regularly discussed with KL, HV, and EW.

### Member checking

To promote descriptive and interpretative validity [37], a written summary of each interview was sent to participants by mail shortly after each interview took place. On one occasion, a participant felt she was misinterpreted during an interview. This was discussed with the participant, and taken into account during data analysis. Furthermore, to promote theoretical validity [37], additional home visits were made to participants, in which the sole purpose was to share our interpretations of the data (after *t*_3_, in June and July 2015). With participants, we discussed findings that were specific to them, including usage patterns and changes we observed during the study. Out of the 33 participants, 25 participated in this final member check. Reasons for not participating were: personal health problems (*n* = 3), deceased (*n* = 3), and lost contact (*n* = 2). Participants recognized themselves very well in our descriptions of them and their use of technologies.

## Results

### Sample

The sample consisted of 33 participants. Nearly 61% of the participants was female. The average age of participants was 76.1 ± 3.9 at *t*_*1*_, and 77.5 ± 3.9 at *t*_*3*_. The majority of the participants had attainted secondary education (61%), while 27% attainted no or only primary education, and 12% attained a form of higher education. During the study, the proportion of participants that lived alone increased from nearly 64% at *t*_*1*_ to 67% at *t*_*2*_ and *t*_*3*_. A proportion of participants received home care: at *t*_*1*_ this was 58%, at *t*_*2*_ nearly 67%, and at *t*_*3*_ nearly 64%. Looking at subjective health; close to 70% of the participants considered their health good, very good, or excellent at *t*_*1*_ and *t*_*2*_. At *t*_*3,*_ this was 61%. Participants’ frailty (TFI) score, was lowest at *t*_*2*_ (3.8 ± 2.4) and highest at *t*_*3*_ (4.6 ± 2.6).^1^ The cognitive functioning (MMSE) score was lowest at *t*_*1*_ (28.1 ± 1.5) and highest at *t*_*2*_ (28.5 ± 1.5).^2^

### Results of the thematic analysis

Thematic analysis of technology usage patterns by participants resulted in two major themes: ‘stable use of technologies’ and ‘shifts to other states of use’. In the following paragraph, we present factors that were related to stable use. In the paragraph immediately after, we describe how stable use was disrupted by various forces that induced shifts to increased, decreased or stopped use. In both paragraphs, longitudinal case descriptions are used to illustrate findings. In the descriptions we use fictive names to protect the identity of participants.

### Stable use of technologies

Analysis of the usage of technologies by the 33 participants showed that the frequency of use of a technology was directly influenced by a combination of six factors: emotional attachment, need compatibility, cues to use, proficiency to use, input of resources and support. Together, these factors formed a system of interrelated components that explained why participants maintained a frequent or less frequent use of certain technologies over time.

**Fig. 1:**
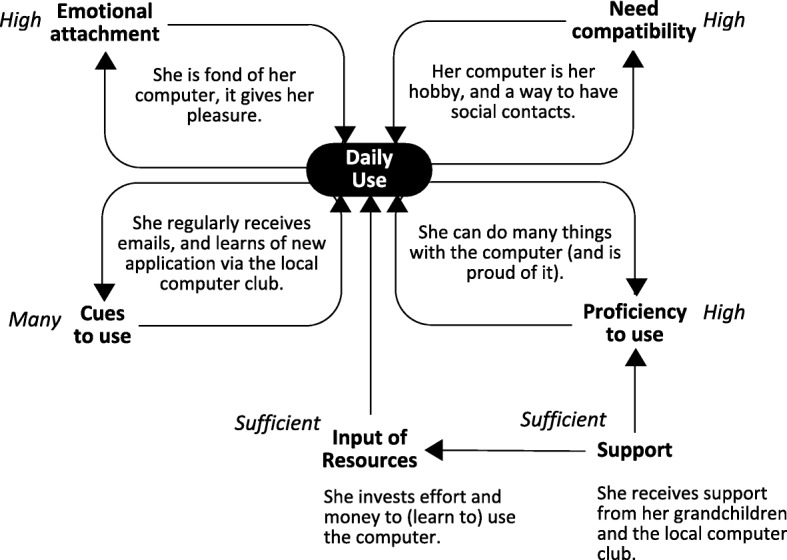
Reasons for Elisabeth’s stable and frequent use of her computer (Templates of figures in this article can be obtained by contacting the first author)

**Fig. 2:**
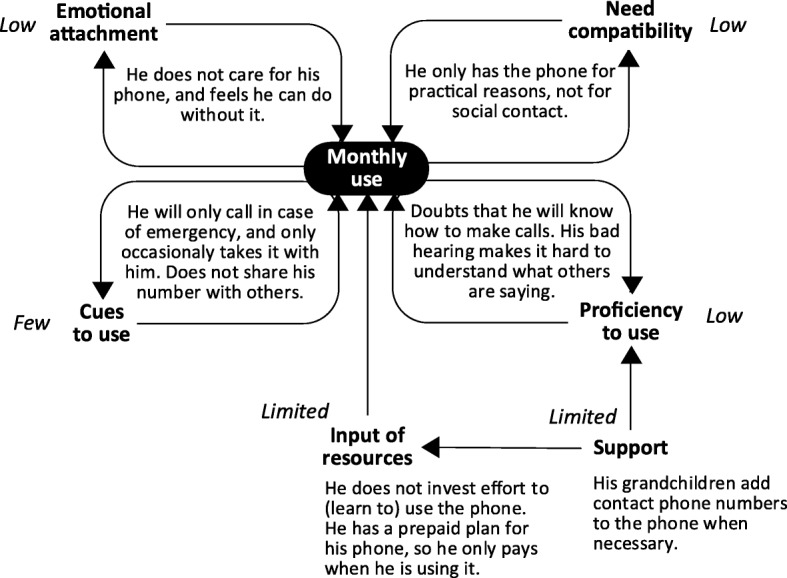
Reasons for Paul’s stable and infrequent use of his mobile phone

Two examples of how this system can operate are displayed in Figs. 1 and 2. Figure 1 explains Elisabeth’s frequent (daily) use of her computer, and Fig. 2 explains Paul’s infrequent (monthly) use of his mobile phone. As can be seen in both figures, frequency of use was influenced by four feedback loops (with emotional attachment, need compatibility, cues to use, and proficiency to use), and two additional factors (input of resources and support). In both cases use was stable, meaning the same frequency of use was reported at *t*_*1,*_ *t*_*2*_ and *t*_*3*_.

In the case of Elisabeth’s frequent use of her computer (Fig. 1), interview data showed that she used her computer because she was emotionally attached to it, and that using the computer cultivated her emotional attachment (hence the feedback loop). As she explained it: *“You can do all sorts of things with it, my music is on it, the photos I take are on it. It’s a lot of fun*” (P20). And: “*I feel like I could be getting addicted to it”* (P20). Elisabeth also used her computer because it was compatible with her needs, and using the computer reaffirmed this (the second feedback loop): “*I: What do you like most about it? P: Just the fact that I am able to send messages and have social contacts. It’s just great! I: You strike me as a social person. P: Yes I definitely am”* (P20). Additionally, Elisabeth experienced certain cues that led her to using the computer. In general, we found that in participants’ lives cues to use could entail specific situations, routines and places. In Elisabeth’s case, she regularly received e-mails because she used her computer to send e-mails (the third feedback loop). Additionally, Elisabeth stated that she used her computer because she learned of new applications at the local computer club, and she went to the computer club because she was a user of the computer. Using the computer also made her feel very proficient, and her proficiency enabled her to make use of the computer (the fourth and last feedback loop): *“It’s good for my self-esteem, the fact that I am able to do it*” (P20). In Elisabeth’s case, there were sufficient resources (i.e., effort and money) to be able to use the computer. These resources were invested directly by herself, and indirectly by external sources of support. As external sources of support she mentioned members of the local computer club and her grandchildren, who also helped her when needed.

In contrast, looking at Paul’s infrequent use of his mobile phone (Fig. 2), data showed that circumstances for technology use were far less favorable. In contrast to Elisabeth’s fondness of her computer, Paul did not care for his mobile phone (low emotional attachment). Additionally, need compatibility was low, since the mobile phone was only in line with one need*: “I only have it for when I go driving, in case the car breaks down*” (P12). This was different from Elisabeth’s case, where the computer was compatible with more of her needs. In Paul’s case, there were also few cues to use, and proficiency to use was low. Lastly, input of resources and external support were both limited. It is important to note that Paul did maintain a certain (infrequent) level of use. However, as a result of him frequently not taking his mobile phone with him, he was not able to call for help when he experienced a fall outside his home. While he regretted not taking it with him, this incident did not affect Pauls’ mobile phone use.

**Fig. 3:**
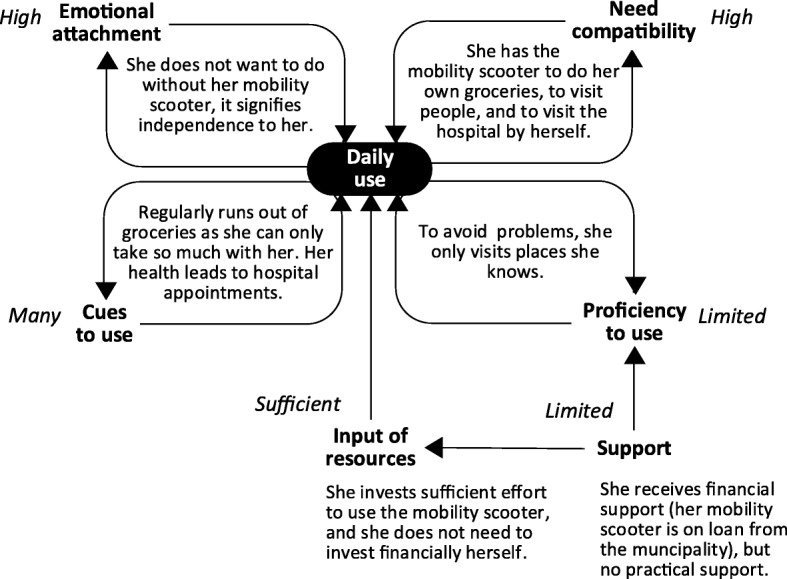
Reasons for Linda’s stable and frequent use of her mobility scooter

Elisabeth’s and Paul’s cases represent two extremes, featuring only favorable or only unfavorable factors influencing technology use. In other less extreme cases, some factors were favorable for technology use, while others were not. An example is displayed in Fig. 3. As seen in this example, Linda used her mobility scooter daily, and need compatibility and emotional attachment were high. There were also many cues to use, and sufficient input of resources. However, Linda’s proficiency to use the mobility scooter was limited as she only felt confident in using the mobility scooter to visit places she already knew. “*I need to know beforehand where I can go, and how to get here. I need to know that*” (P28). As a result, she was dependent on a local bus service for people with disabilities, if she wanted to visit a place that was new to her: “*Then I need to make use of the special bus service … it requires you to make an advanced reservation... when you want to go back home, you stand there and wait”* (P28).

### Shifts to other states of use

**Fig. 4:**
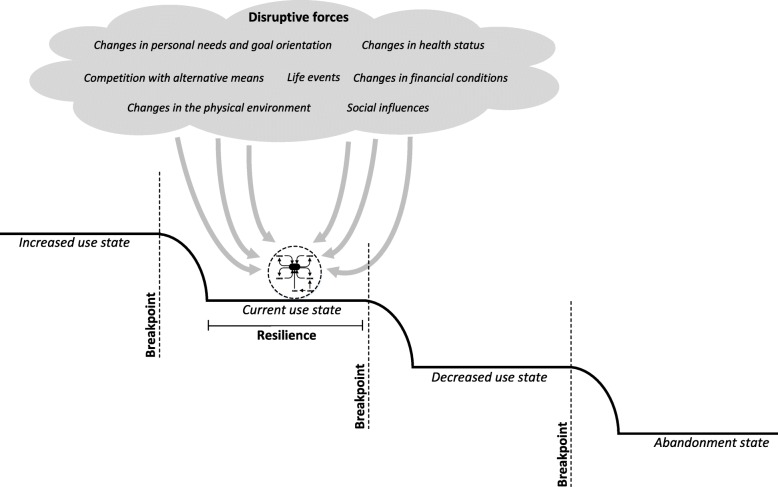
Shifts to other states of use as a result of disruptive forces

Longitudinal analysis showed that the use of technologies by the 33 participants was subject to disruptive forces (Fig. 4). These forces could influence the six interrelated factors that were described in the previous paragraph. As a consequence of these dynamics, the use of a technology at a certain point in time (i.e., the current use state), could change to a state of increased use or decreased use. Dynamics between disruptive forces and the system of the six interrelated factors could also lead to a situation in which a participant stopped using a technology (i.e., the abandonment state). Additionally, it appeared that a certain amount of disruption had to take place before use actually changed to a different state. In other words, there were breakpoints. Moreover, the use of a technology was in some cases more resilient to disruption than in other cases. This depended on the robustness of the system of the six interrelated factors (e.g., the level of emotional attachment, the amount of cues to use), and on how quickly and effectively participants and external sources of support responded to disruption. Personal characteristics of participants played a role here (including an active vs. a passive coping style, willingness to change, willingness to ask for support).

**Fig. 5:**
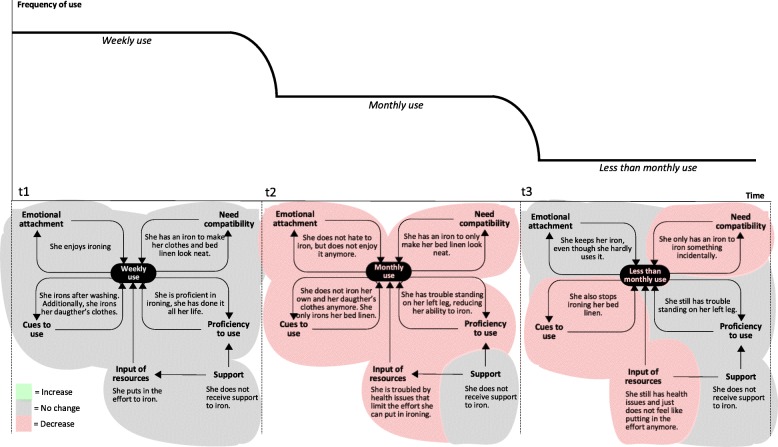
Sheila’s use of her iron at time points at t_1,_ t_2_ and t_3_

Analysis showed that the abovementioned dynamics applied to the wide range of technologies that were used by participants (including home appliances, telephones and ICT and assistive devices). For example, Fig. 5 displays Sheila’s gradually decreased use of an appliance that she had used for the most part of her life: an iron. When we visited Sheila at *t*_1_ she used her iron every week. At that time, she rather enjoyed ironing and also ironed clothes of her daughter. At *t*_2_, things had changed considerably. She had experienced a fall, and her arthrosis bothered her more than before: *“I cannot stand so long on my legs anymore, particularly my left leg”* (P13). These changes in her health status had several effects: she could not iron as much as before (lower proficiency to use, and less investment of resources), she did not enjoy ironing as much as before (lower emotional attachment), and she ironed in less situations (less cues to use). Additionally, Sheila still wanted to keep her clothes tidy but she could not use her iron to meet this need anymore (lower need compatibility). Instead, she used alternatives to ironing, such as hanging and folding her clothes. This also occurred in cases which involved other participants: decreased use of a technology could go together with increased use of alternatives to that technology. Sheila used alternative means because she was forced to, because she could not iron anymore. In contrast, we also saw cases in which participants voluntary decided to make more use of an alternative mean to meet their needs. In Sheila’s case, the result of the abovementioned developments was a notable decline in frequency of use at *t*_2_. Frequency of use continued to decline, and at *t*_3_ she only used her iron incidentally. At this stage, Sheila still had health issues, although her legs had not gotten worse. It seemed that Sheila had come to terms with hardly using the iron *“I am at a point where I do not care anymore about it … No, I just don’t feel like using it”* (P13). We saw this more often among participants; it seemed like there was a point in which they had gotten used to the new state of affairs. This also occurred in some cases in which the use of a technology was temporarily decreased due to a life event (e.g., a partner having a serious illness) or less dramatic events such as getting the flu or temporarily receiving less support. After a while, need compatibility, usage cues and emotional attachment would decline, as participants realized they could very well live with using the technology less. If use was decreased long enough, this could ultimately lead to stopped use (i.e., abandonment state). In particular, this was the case when use had become so infrequent that proficiency became severely impeded, as one participant puts it *“I cannot work on it (the computer) anymore. That would mean that I would have to learn it all over again”* (P2). In other cases, participants primarily stopped using a technology because their needs (or priorities) had changed. For example, a participant who previously had used a home alarm system for security reasons *“Now it is not necessary anymore, I am always at home. When I bought it, I used to still go on vacation regularly”* (P14). Going back to Sheila’s case: although she hardly ever used her iron, she still kept it in her home. In general, we found that participants had a tendency to hold on to devices that they seldom used or had stopped using completely: *“I was born in 1937, I am not used to throwing things away*” (P15). When a device did leave the home, this was usually because it had broken down and was replaced, or because a family member expressed interest in using it.

**Fig. 6:**
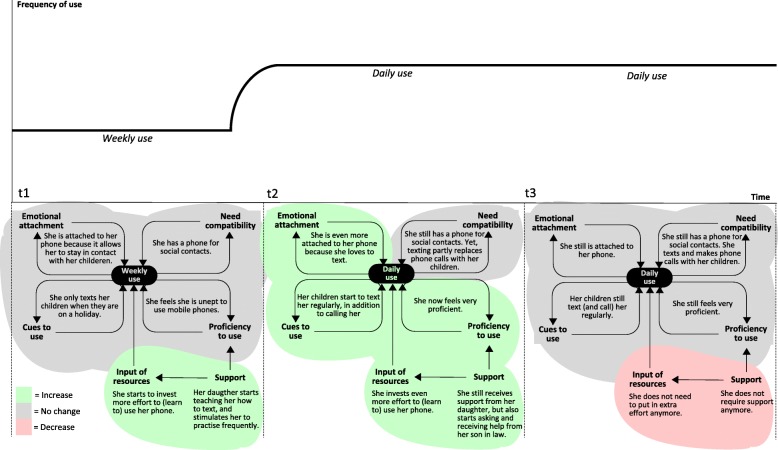
Elly’s use of her mobile phone at time points at t_1,_ t_2_ and t_3_

In contrast to the abovementioned, an example of increased use is displayed in Fig. 6. When we first visited Elly, she used her mobile phone on a weekly basis. She only used it to make telephone calls and did not feel proficient to do anything else with it. However, her daughter had started to teach her how to send text messages, and she encouraged Elly to practice regularly, which she did. The support that Elly received from her children was the result of a recent life event. Just prior to participating in the study, she had lost her husband which meant that she was *“on her own”*, and this had motivated her children to help her more. At *t*_2,_ Elly’s use of her mobile phone had gone from weekly to daily. By that time, Elly felt very proficient in using her mobile phone and was proud of it: *“It may sound crazy, but I consider it a victory”*(P30). There were many cues for her to text with her mobile phone: *“The children all do it. I get a message on my phone, I read it, and quickly send a message back. A quick reply, and I receive another one, and I reply again!”* (P30). She was also more emotionally attached to using her phone: “*I feel I do not want to miss these messages*” (P30). The aforementioned chain of events occurred often in cases in which the use of a multifunctional device (mostly ICT) increased. In these cases, increased use was induced and supported by the social network. When we visited Elly at *t*_3,_ she still used her mobile phone daily. By that time, she was so familiar with her phone that using it was effortless, and she did not need support anymore. However, Elly had gotten a tablet computer from her children just before *t*_3._ In fact, she started to prefer the tablet over her mobile phone when it came to sending text messages*: “I still use my phone and using it is easy. But I feel that typing on my tablet is more convenient”* (P30), and: *“The tablet is new, but it is actually starting to replace my phone”* (P30). According to Elly, she felt confident that she could use the tablet because of her positive experiences in learning to use her mobile phone. While Elly’s case is an example of positive developments leading to an increased use state, there could also be negative or less favorable developments that increased use. One example is decreased health leading to the increased use of assistive technologies. Another example is the disappearance of alternatives to a technology. There was a participant (a widower) who had the habit of eating dinner at his son’s house, who was unemployed. The participant described himself as *“not the cooking type”* (P25). This situation changed when his son and his son’s wife both got a job. He was now forced to cook considerably more, and did this by making much more use of his microwave oven (for preparing microwave meals). His microwave oven became essential to him: “*I can’t do without it. How else am I supposed to prepare meals?”* (P25).

## Discussion

**Fig. 7:**
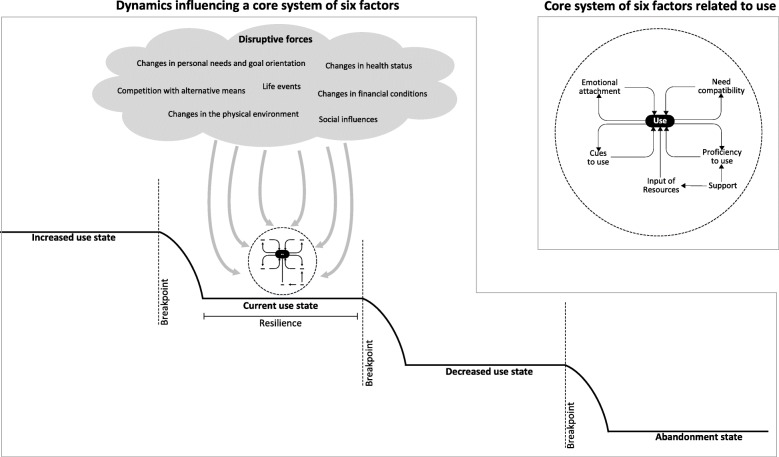
Dynamics In Technology Use by Seniors (the DITUS framework)

The current study sought to explain changes and stability in the use of technologies by independent-living seniors over time. Findings and described concepts are summarized in a new dynamical framework that is presented in Fig. 7: Dynamics In Technology Use by Seniors (DITUS). Results showed that the developed DITUS framework was effective in explaining the use of technologies by participants over a period of one and three quarter years. We found that there was a core of six interrelated factors that were closely linked to the frequency of technology use: emotional attachment, need compatibility, cues to use, proficiency to use, input of resources, and support. Additionally, there were disruptive forces that could induce changes to other levels of use by affecting these six factors. Disruptive forces included: social influences, competition with alternative means, changes of personal needs and goal orientation, changes in health status, changes in the physical environment, and changes of financial conditions. Whether or not disruptive forces induced change was dependent on how strong they were, on how long they acted, and on the level of resilience to change. The latter mainly depended on the state of the core of six factors in the first place, and on how quickly and effectively participants and external sources of support responded to disruption. Our results also showed there was overlap between the use of technologies; multiple technologies could address the same needs, proficiency to use could affect multiple technologies, and multiple technologies could tap into the same pool of internal resources and external support. Additionally, the use of multiple technologies could be interrelated because cues to use (specific situations, routines and places inducing use) were linked.

In the literature, there is a lack of longitudinal research on consumers’ use of technologies that have been accepted into the home (i.e., post-implementation acceptance) [38, 39]. This research gap is also reflected in slow theoretical development concerning long-term use. More specifically, most existing technology acceptance models are solely focused at predicting pre-implementation acceptance (i.e., reasons for starting to use a technology in the first place). This includes (various versions of) the Technology Acceptance Model (TAM) [40–42], the Unified Theory of Acceptance and Use of Technology (UTAUT) [43, 44], and the Senior Technology Acceptance Model (STAM) by Renaud and van Biljon [45] and Chen and Chan [46]. Looking at established technology acceptance models, only the Information Systems Continuance model (ISCM) specifically considers post-implementation acceptance [13, 47]. It suggests that the perceived usefulness of a technology is more crucial for pre-implementation acceptance, and that satisfaction with the technology is more dominant for post-implementation acceptance. However, ISCM was not developed with older adults in mind, which might explain why it does not address the full range of factors and dynamics that are described in this study. Furthermore, ISCM focuses on the intention to continue use, while DITUS focuses on actual use behavior.

Our findings are more in line with a five-week ethnographic study of experiences of young adults who purchased and used an Apple iPhone for the first time [39]. The authors of this study found that functional dependency, emotional attachment and familiarity were most important in participants’ experiences with the technology. They also found that ease of use became less of a concern to participants over time [39]. This however differs from the current study, in which we found that - for older adults- the proficiency to use a technology remains crucial, and that external support can play an important role in this respect. The latter is in line with a recent longitudinal study on older adults’ use of mobile ICT devices [48]. The authors of the iPhone study also found that negative experiences with the technology seemed to become less relevant to users’ satisfaction as time progressed. We observed a similar pattern, in the sense that negative experiences were sometimes reported by participants, but did not seem to be influential in explaining use.

It is important to note that our findings are aligned with aspects of Rogers’ Diffusion Of Innovations (DOI) theory [49]. Our concept of need compatibility is in large part identical to Roger’s concept of compatibility. Additionally, Rogers theory also illuminates to the prominent role of both social influences and competing alternatives in the use of technologies by individuals. While being a highly influential theory, DOI is primarily focused on pre-implementation acceptance [13, 49]. The incorporation of post-implementation acceptance is limited to the so called “confirmation stage”, in which an individual seeks reinforcement for the already made decision to start using a technology. The outcome of this stage can be discontinuance, meaning the decision to reject a technology after it has previously been used. As such, DOI’s consideration of long-term use is limited to post-implementation beliefs and does not take into account other aspects of post-implementation acceptance, including changing circumstances and motivations for use.

Seeing that most older adults will go through cognitive, physical and social changes as they age, one could argue that there is a great need for more longitudinal post-implementation research among this target group. In this paper, we have presented a framework for studying technology use dynamics that can be helpful in this pursuit. The strength of the current framework is that it is dynamical minimalist, meaning it is parsimonious without losing depth of understanding [50]. By forming a dynamical model, we believe we were able to identify the simplest mechanisms and fewest variables capable of producing the complex phenomenon in question (i.e., technology use over time by independent-living seniors). Another strength of the framework is that it can be linked to other theories or phenomena. For example, research on how technology use is influenced by the onset and progression of dementia. In terms of our framework, dementia is considered a disruptive force that is expected to influence several of the core of six factors in the framework. It would be interesting to understand which of these factors are affected to which extent (and for how long), which of these factors could possibly compensate for decline in other factors, and how different levels and types of resilience may buffer the effects of dementia on technology use. This could complement previous work on peoples’ everyday use of technology while experiencing dementia [51, 52]. Additionally, it could be worthwhile to explore links between the DITUS framework and theories of successful aging, such as the Selective Optimization with Compensation model (SOC-model) [17, 53]. According to the SOC-model, successful aging is an ongoing and dynamic process in which three processes play an important role: people’s Selection of life domains that are important to them, Optimization of means and resources that facilitate success in these domains, and Compensation for losses in these domains [53]. We have observed in our data that the process of selection (i.e., changes in personal needs and goal orientation) can disrupt the current state of use of a technology. With regards to the compensation process: our findings indicate that there can be competition between means that could compensate for losses in domains. Lastly, our findings show that the capacity to optimize of the use of technological means is dependent on actions and coping style of both participants and external sources of support (i.e., resilience).

Several study limitations need to be noted. First, while our framework allowed us to explain and describe the phenomena in our data, this does not mean that our findings are exhaustive. Older adults may experience other (combinations of) disruptive forces than our participants, and these may affect the core six variables in ways we have not encountered. Furthermore, we have only been able to collect data on technology products that were in the homes of our participants at the time of our study. Since our data collection, new technologies have entered the marketplace. Additional studies in other populations are necessary to determine to what extent our results and framework can be transferred to other contexts and apply to newer technologies.

Second, some limitations are related to the application of DST. For example, from DST we know that some variables in a dynamical system may fluctuate more quickly than others [24]. Additionally, change is not always proportional to input, meaning small changes can have a dramatic effect on outcomes, or large changes can have a modest effect [21]. Furthermore, feedback loops may not only influence the outcome directly, but may also influence each other [24]. These issues can be addressed better by quantitative empirical testing of the proposed DITUS framework.

Third, potential biases of our study need to be discussed. Our findings could be susceptible to recall bias, since the interviews were in part retrospective. Additionally, our findings are affected and possibly biased by our beliefs, values, and assumptions. We addressed this by working in alternating pairs during data collection and analysis, and by critically evaluating the design and findings in group discussions involving all the authors. It is important to note that the current study is focused on understanding the micro-level (i.e., technology use by the individual). Our results highlight the influence of the meso level (i.e., the social network) on the micro level. However, this is only studied unidirectionally (i.e., we did not investigate how the micro level influences the meso level). Additionally, the macro (societal) level is beyond the scope of the current study. As such, multi-level socio-technical research on long-term technology use by older adults may yield crucial additional insights [54, 55].

Recently, it has been argued to define people’s health as ‘*the ability to adapt and self-manage in the face of social, physical and emotional challenges*’ [56]. This implicates that technological solutions that aim to support aging in place should (a) be able to adapt to changes that people go through, or (b) be robust in the sense that they can still be used effectively while facing changes, and (c) be capable of mitigating unfavorable changes. To improve sustainability, technological solutions and services can promote three interrelated levels: motivations for use (emotional attachment and need compatibility), opportunities to use (cues to use and proficiency to use), and resources to use (input of resources and support). Additionally, technological solutions, and the people who design and implement them, need to gain understanding on how favorable and unfavorable disruptions influence the aforementioned levels.

## Conclusions

Aging is complex, dynamic and personal, and this is also reflected in the use of technologies by older people. Periods of stability and periods of change both occur naturally. The DITUS framework can aid in understanding stability and instability in long-term technology use, and in developing and implementing sustainable technological solutions for aging in place.

## Additional file

Additional file 1: Inteview guide. (DOCX 21 kb)

## Data Availability

Anonymized analyses of technology usage patterns are available from the corresponding author on reasonable request. Raw data (interview transcripts) cannot be publicly released do to the risk the respondent confidentiality will be compromised.

## Abbreviations

DITUS: Dynamics In Technology Use by Seniors
DST: Dynamical Systems Theory
MMSE: Mini-Mental State Examination
SOC-model: Selective Optimization with Compensation model
TFI: Tilburg Frailty Indicator

## Footnotes

1 As suggested by Gobbens et al. [32] a Tilburg Frailty Indicator (TFI) score of 5 was used as the cut-off point for frailty.

2 As suggested by Kempen, Brilman and Ormel [28] a Mini-Mental State Examination (MMSE) score of 24 was used as the cut-off point for cognitive impairment.
