# Supplementary material for: Understanding changes and stability in the long-term use of technologies by seniors who are aging in place: a dynamical framework
Source: BMC Geriatr. 2019 Aug 28;19:236. doi: 10.1186/s12877-019-1241-9 (PMC6712781; doi:10.1186/s12877-019-1241-9)
Supplement: Supplementary file 1 — Inteview guide. (DOCX 21 kb) [file 12877_2019_1241_MOESM1_ESM.docx]

# Interview guide

For the BMC Geriatrics article *Understanding Changes and Stability in the Use of Technologies by Seniors Who Are Aging in Place: a Dynamical Framework*” (Peek et al., 2019). Translated from Dutch to English by the authors.

## Wave 1

Prior to the semi-structured interview, background information was collected using a short survey and participants and researchers jointly made a tour through the home to create an inventory of the technologies in the home (see the original article).

Next, participants were interviewed on reasons for their frequency of use of three technologies. Which technologies were discussed depended on preferences of the participants (who displayed strong feelings towards certain technologies) and on suggestions by the researchers (who aimed to understand the usage of multiple types of technology). In particular, the researchers aimed to include technologies that were integrated in the daily lives of participants, as well as technologies that were not, or to a lesser extent.

These questions and topics were used during the semi-structured interview:

**Opening question**: Can you explain to me why you are using this technology [mention the frequency of use]?

**Topics related to barriers and facilitators of use**:

- Experienced advantages and disadvantages;
- Stimulating and impeding factors;
- Concerns and doubts.

Regularly check after a participant mentions a topic/reason: Do you feel that this has affected your use of the technology?

**Zooming in on personal characteristics and social influences:**

During our conversation, you have mentioned [personal characteristic or social influence]. Can you tell me more about this? Do you feel that this has affected your use of the technology in any way?

**Specific topics:**

- Perceived need for having and using the technology;
- Extent to which the technology can support independent-living;
- Costs of using the technology;
- The fact that others can see that the person is using/wearing a technology (i.e., stigmatization).

Regularly check after a participant mentions a topic/reason: Do you feel that this has affected your use of the technology?

The abovementioned topics are based on our systematic literature review: Peek STM, Wouters EJM, van Hoof J, Luijkx KG, Boeije HR, Vrijhoef HJM. Factors influencing acceptance of technology for aging in place: A systematic review. Int. J. Med. Inform. 2014;83:235–48.

## Wave 2

Prior to the semi-structured interview, background information and the technology inventory were updated. Next, semi-structured interviews were conducted on at least one technology of which the frequency of use was identical to the previous visit, at least one technology of which use had increased, and at least one technology of which use had decreased or stopped entirely. Again, the focus was understanding the usage of multiple types of technology.

Please note that the researchers studied the coded wave 1 transcripts before they visited the participants and asked the questions below.

Cases in which technology use was increased/decreased, or had stopped:

**Opening question**: The last time I was here, you used this technology [mention the frequency of use]. Now you are using it [mention the frequency of use]. Can you explain to me why?

**Specific topics:**

- Positive and negative experiences;
- Met or unmet expectations;
- The balance between, or changes in advantages and disadvantages;
- Concerns and doubts regarding the technology;
- Changes in personal circumstances (health, limitations, life events);
- Personality traits and habits;
- Alternatives to the technology
- Costs
- Social influences

Do you feel that you need the technology? (explore reasons for and against)

Cases in which use was frequency of use was identical to the previous visit:

**Opening question**: The last time I was here (x months ago), you used this technology

just as frequent as you do now: [mention the frequency of use] . Can you explain to me why?

**Specific topics:**

- Positive and negative experiences;
- Met or unmet expectations;
- The balance between, or changes in advantages and disadvantages;
- Concerns and doubts regarding the technology;
- Changes in personal circumstances (health, limitations, life events);
- Personality traits and habits;
- Alternatives to the technology
- Costs
- Social influences

I would like to ask you about your daily routines and habits.

When do you use the technology? And in what situations?

In cases where use is not linked to routines or habits: Can you tell me why you are not using the technology on a routinely basis?

Do you feel that you need the technology? (explore reasons for and against)

The abovementioned topics and questions are based on our data analysis of the first wave.

## Wave 3

Similar to wave 2, prior to the semi-structured interview, background information and the technology inventory were updated. Next, semi-structured interviews were conducted on at least one technology of which the frequency of use was identical to the previous visit, at least one technology of which use had increased, and at least one technology of which use had decreased or stopped entirely. Again, the focus was understanding the usage of multiple types of technology.

Please note that the researchers studied the coded wave 2 transcripts before they visited the participants and asked the questions below.

In all cases:

**Opening question**: The last time I was here, you used this technology [mention the frequency of use]. Now you are using it [mention the frequency of use]. Can you explain to me why?

**Technology topics:**

- Positive and negative consequences of using the technology;
- Positive and negative properties of the technology;
- Perceived need for using the technology.

**Usage topics:**

- Ease of use;
- Effort required to use;
- Asking/receiving support;
- Routines and habits.

**Personal characteristics topics:**

- Needs;
- Health;
- Life events;
- Technological experience and self-confidence;
- Interest in technology;
- Willingness to invest in technology.

**Contextual topics:**

- Social and societal influences;
- Alternatives to the technology;
- Influence of the technology supplier;
- Financial factors;
- Time / Place / Weather;

Extra for cases in which use was frequency of use was identical to the previous visit:

When do you use the technology? And in what situations?

In cases where use is not linked to routines or habits: Can you tell me why you are not using the technology on a routinely basis?

The abovementioned topics and questions are based on our data analysis of the second wave.
